# Structure and magnetic properties of icosahedral Pd_x_Ag_13−x_ (x = 0–13) clusters

**DOI:** 10.1038/s41598-017-10184-6

**Published:** 2017-08-25

**Authors:** Bai Fan, Gui-Xian Ge, Cheng-Huan Jiang, Guang-Hou Wang, Jian-guo Wan

**Affiliations:** 1 National Laboratory of Solid State Microstructures, and Department of Physics, Nanjing University, Nanjing, 210093 China; 2 Key Laboratory of Ecophysics and Department of Physics, College of Science, Shihezi University, Xinjiang, 832003 China; 3 Collaborative Innovation Center of Advanced Microstructures, Nanjing University, Nanjing, 210093 China

## Abstract

In this article, we present a modified Velocity-Verlet algorithm that makes cluster system converge rapidly and accurately. By combining it with molecular dynamics simulations, we develop an effective global sampling method for extracting isomers of bimetallic clusters. Using this method, we obtain the isomers of icosahedral Pd_x_Ag_13−x_ (x = 0–13). Additionally, using the first-principle spin-polarized density functional theory approach, we find that each isomer still retains its icosahedral structure because of strong *s*-*d* orbital hybridization, and the cluster is more stable when a Pd atom is at the center of the cluster. With increasing x value, the magnetic moment decreases linearly from 5.0 μB at x = 0, until reaching zero at x = 5, and then increases linearly up to 8.0 μB at x = 13. By calculating the atom-projected density of states (PDOS), we reveal that the magnetic moment of Pd_x_Ag_13−x_ mainly originates from *s* electrons of Ag when 0 ≤ x < 5, and *d* electrons of Pd when 5 < x ≤ 13. The PDOS results also show that the Pd_x_Ag_13−x_ tends to transform from a semiconductor state to semi-metallic state when x gradually increases from 0 to 13.

## Introduction

Bimetallic clusters have received much attention due to their unique optical, magnetic and catalytic properties^1, 2^. The properties of bimetallic clusters depend on their structure to a great extent^3, 4^. For instance, stable structures of Co_18-m_Cu_m_ (0 ≤ m ≤ 18) clusters show evident segregation phenomenon, and their magnetic moment varies with elemental composition^5^. The doping of one Mn atom can enhance the stability of Pd_n_ (n = 3–19) clusters, and their magnetic moment can also be increased by a magnitude of 3–5 μB^6^. Searching for bimetallic clusters with good stability and property is now becoming a hot topic in the field of clusters due to their potential important applications.

Searching for stable structures is actually a rather difficult task due to the structural complexity of mixed clusters. Constructing mixed clusters with magic structures are regarded as an effective avenue. In this work, we focus on the study of stable structures of Pd-Ag bimetallic clusters as well as their magnetic properties. Previous studies have shown that both Pd clusters and Ag clusters exhibit magnetic moments, nevertheless, silver clusters have weak magnetic moment, e.g., less than 2.0 μB for Ag_n_ (n ≤ 12) and 5.0 μB for Ag_13_, while Pd_n_ (n = 3–19) clusters exhibit increased magnetic moments in stepwise manner with increasing n^4–8^. Different from single-elemental clusters, the structures of Pd-Ag clusters are more complex, so are the origins of magnetic moments. Some theoretical methods have been used to calculate the structures of Pd-Ag clusters, such as an adaptive immune optimization algorithm^9^, molecular dynamics simulations^10^, and a modified basin hopping Monte Carlo simulation^2^. All these studies have pointed out that when Pd atoms aggregate at the center of the cluster and Ag atoms segregate to the surface, the Pd-Ag clusters are more stable.

An effective global search approach for extracting isomers of bimetallic clusters is crucial for investigating their properties. In previous research, a genetic algorithm was usually employed to search for stable structures of bimetallic clusters. Genetic algorithm is inspired on the Darwinian evolution process, which was first developed by Deaven *et al*.^11^. To obtain more isomers, the number of individuals of population should be enlarged as much as possible, but it also increases the computational time. Moreover, isomers of bimetallic clusters increase rapidly with increasing cluster size. For a A_m_B_n_ bimetallic cluster with a given structure^12^, it has geometrical arrangements as many as (n + m)!/(n!m!). Extracting isomers from such a large number of clusters corresponding to all (n + m)!/(n!m!) geometrical arrangements is crucial to further study the properties of bimetallic clusters.

Molecular dynamic simulation is another effective method to search for stable structures of bimetallic clusters. In previous studies, the standard Velocity-Verlet algorithm was usually used during the molecular dynamic simulation, however, the main drawback of this algorithm is that the energy convergence is hard to be achieved and the energy convergence accuracy is also not high, which makes it difficult to obtain all possible isomers. Herein, we propose a modified standard Velocity-Verlet algorithm combined with molecular dynamics simulations, in order to quickly and accurately solve the energy convergence for optimizing the bimetallic cluster structure. On this basis, we develop an effective global sampling method for extracting isomers of bimetallic clusters. Using this method, we obtain the isomers of icosahedral Pd_x_Ag_13−x_ (x = 0–13) clusters. We further optimize these structures and calculate their magnetic properties using the first-principle spin-polarized density functional theory (DFT) approach. The results show that all the isomers of Pd_x_Ag_13−x_ retain well the icosahedron structure, and their magnetic moment exhibits regular change with elemental composition. Moreover, based on the calculations of the atom-projected density of states (PDOS) of Ag and Pd in the clusters, we provide an insight into the origin of the magnetic moments of Pd_x_Ag_13−x_.

## Results and Discussion

### Structure and electronic properties

**Figure 1:**
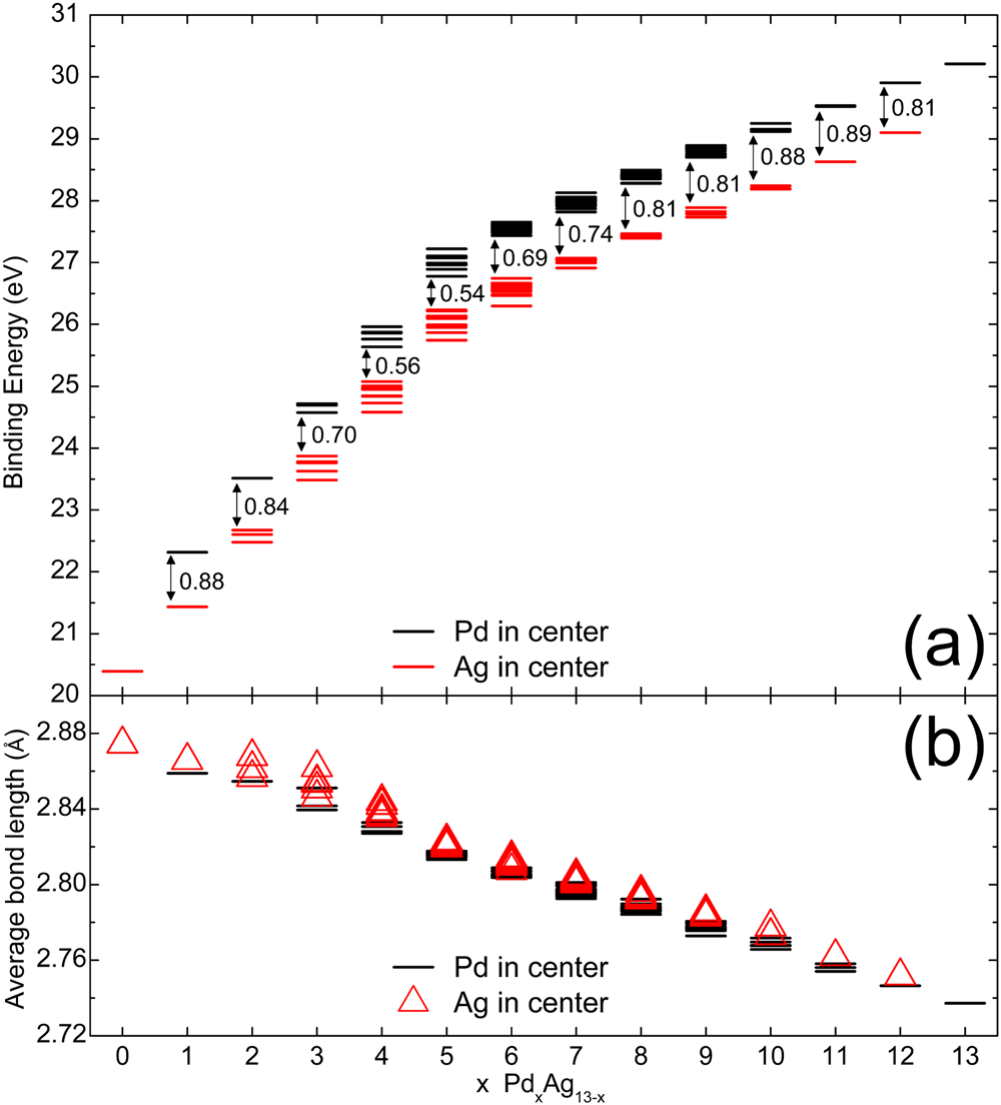
(**a**) Binding energy (eV) of Pd_x_Ag_13−x_ (x = 0–13) clusters from DFT calculations, where the value near the double headed arrow is the binding energy difference (ΔE_b_) for the corresponding x value, respectively. (**b**) Average bond length (Å) of the clusters.

In order to investigate the stability of the Pd_x_Ag_13−x_ (x = 0–13) cluster, we calculated its binding energy, which is defined as:

1 $${{\rm{E}}}_{{\rm{b}}}={\rm{xE}}({\rm{Pd}})+(13-{\rm{x}}){\rm{E}}({\rm{Ag}})-{\rm{E}}({{\rm{Pd}}}_{{\rm{x}}}{{\rm{Ag}}}_{13-{\rm{x}}})$$

where E(Pd), E(Ag) and E(Pd_x_Ag_13−x_) are the energy of the Pd atom, Ag atom and Pd_x_Ag_13−x_ cluster, respectively. After extracting the icosahedral Pd_x_Ag_13−x_ clusters, we obtained 164 clusters, half of which are clusters with a central Pd atom. All the icosahedral Pd_x_Ag_13−x_ clusters still retain the icosahedron structure after DFT calculations. The binding energies of all the Pd_x_Ag_13−x_ after DFT calculations are shown in Fig. 1(a). It is evident that when the Pd atom is at the center of the cluster, the binding energy is higher, indicating that the cluster is more stable. Here, we define the binding energy difference (ΔE_b_) of Pd_x_Ag_13−x_ as the difference between the minimum binding energy of the clusters with a central Pd atom and the maximum binding energy of the clusters with a central Ag atom, which is also shown in Fig. 1(a). Noteworthy, the ΔE_b_ value varies with x, and the smallest ΔE_b_ value, 0.54 eV, appears at x = 5. The results shown in Fig. 1(b) reveal that the average bond length of Pd_x_Ag_13−x_ decreases with increasing x value. The average bond length is shorter when the Pd atom is at the center of the cluster, indicating that the interaction between atoms is stronger.

**Figure 2:**
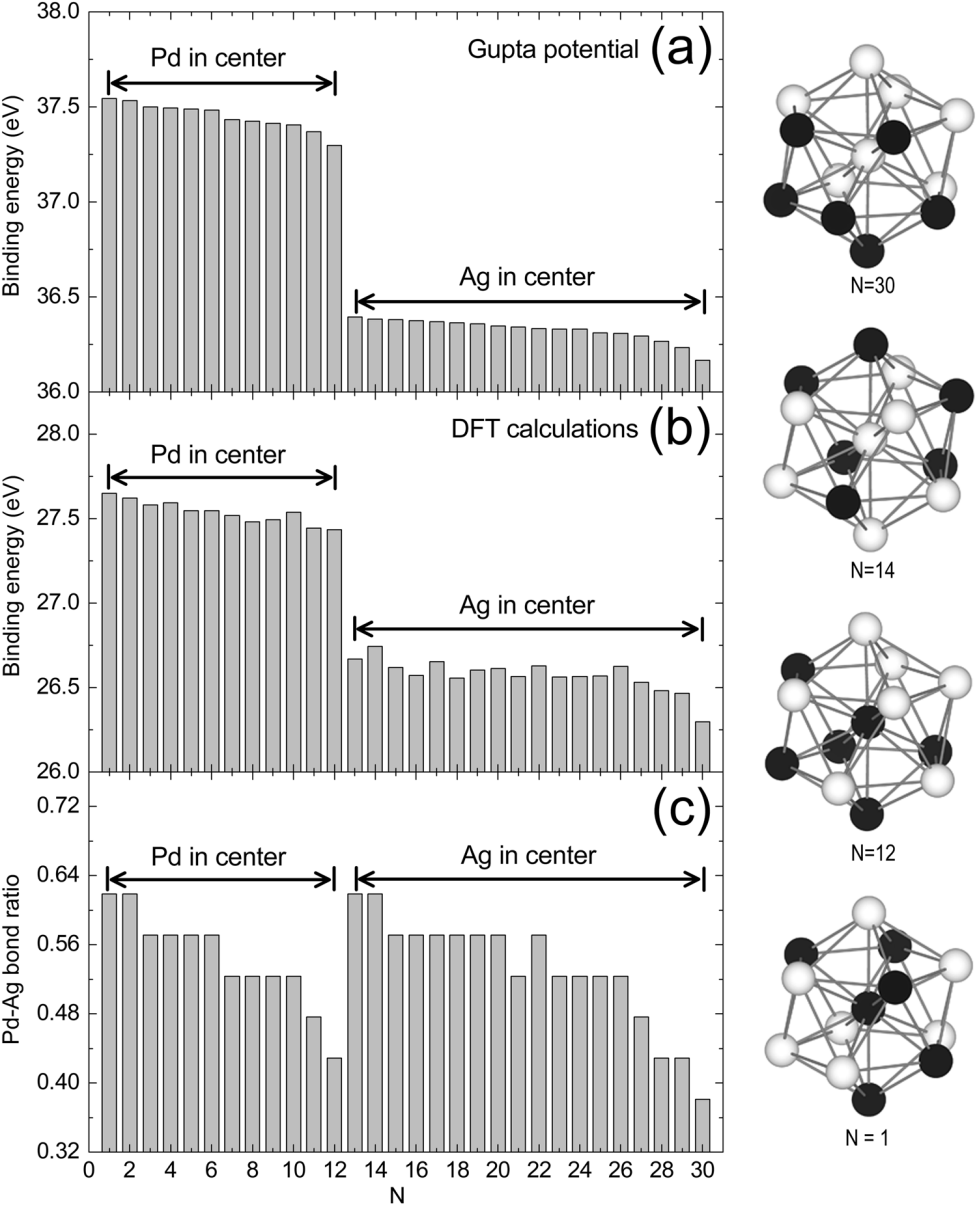
The black and white spheres represent the Pd and Ag atoms, respectively. N is the ordinal of the Pd_6_Ag_7_ clusters with the decreasing binding energy after molecular dynamics simulations with the Gupta potential. (**a**) Binding energy (eV) of the Pd_6_Ag_7_ clusters after molecular dynamics simulations with the Gupta potential. (**b**) Binding energy (eV) of the Pd_6_Ag_7_ clusters from the DFT calculations. (**c**) Pd-Ag bond ratio of the Pd_6_Ag_7_ clusters.

To facilitate the following analysis, we define the A-B bond ratio in the cluster as the ratio of the A-B bond number to the total bond number (A = Pd, Ag; B = Pd, Ag). Evidently, the larger the Pd-Ag bond ratio is, the greater the degree of mixing of the two kinds of atoms is. To better understand the structure characteristics of the icosahedral Pd_x_Ag_13−x_ clusters, we take Pd_6_Ag_7_ as an example to conduct a detailed analysis. Pd_x_Ag_13−x_ has 30 isomers in total, and the binding energy and Pd-Ag bond ratio of all these isomers are compared in Fig. 2(a)–(c).

According to the value of the binding energy predicted from the Gupta potential, we labeled these isomers using the parameter N. A larger N value means that the binding energy is small. The data in Fig. 2(a)–(c) reveal that the clusters with a central Pd atom (N = 1–12) have much larger binding energy than the clusters with a central Ag atom (N = 13–30), indicating higher stability. As shown in Fig. 2(c), the Pd-Ag bond ratio increases with increasing binding energy, whether the atom at the center of the cluster is a Pd or Ag atom. This indicates that the cluster shows high stability if the Pd-Ag bond ratio becomes large.

**Figure 3:**
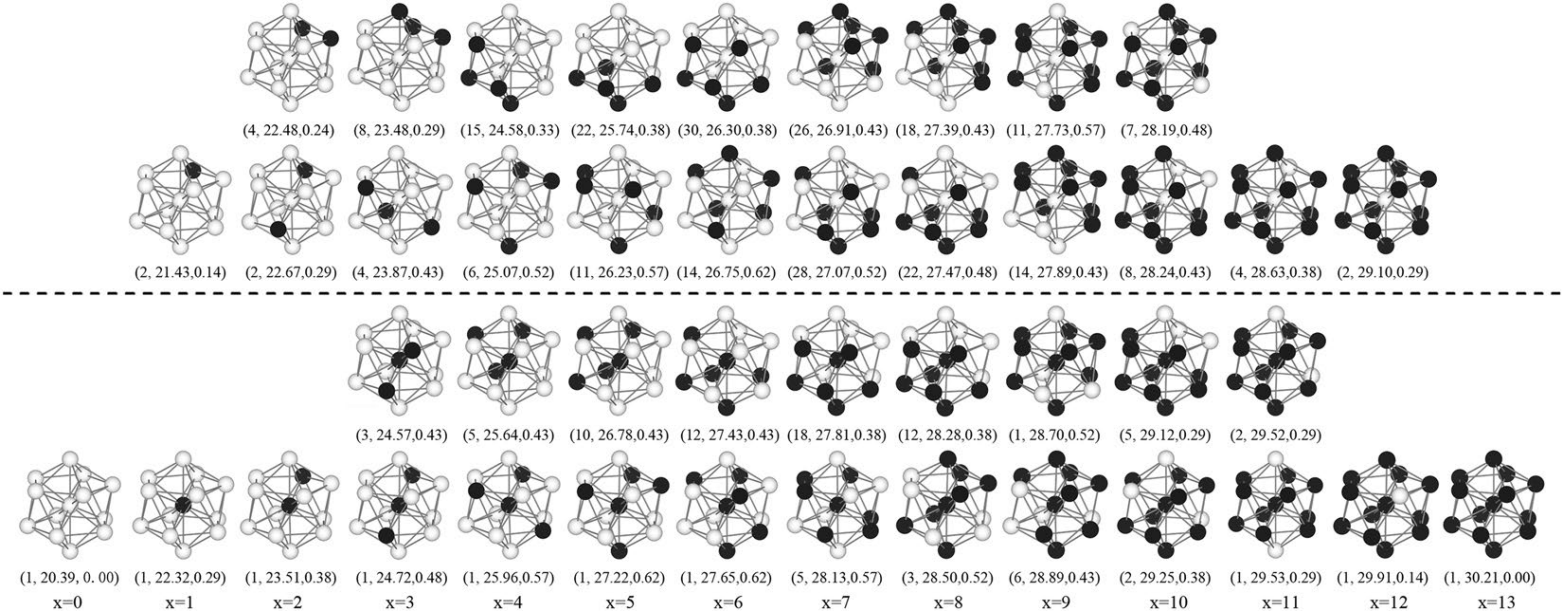
Structures of the Pd_x_Ag_13−x_ (x = 0–13) clusters, black and white spheres represent the Pd and Ag atoms, respectively. The two typical clusters with central Pd atom are underlined with black dashes, and have maximal binding energy (down) and minimal binding energy (up). The two typical clusters with a central Ag atom are plotted above the black dashed line, and have maximal binding energy (down) and minimal binding energy (up). For each x value, the first value in the bracket is the ordinal N of Pd_x_Ag_13−x_, the second and third values are the binding energy from the DFT calculations and the Pd-Ag bond ratio, respectively.

The typical geometric structures for all the Pd_x_Ag_13−x_ (x = 1–13), presented in Fig. 3, show that the structure with a central Pd atom has larger binding energy than the structure with a central Ag atom. In addition, for most Ag-Pd clusters, whether the central atom is Pd or Ag, the large Pd-Ag bond ratio is helpful to improve the binding energy. In other word, increasing the degree of mixing of the two kinds of atoms can enhance the stability of the Ag-Pd cluster.

**Figure 4:**
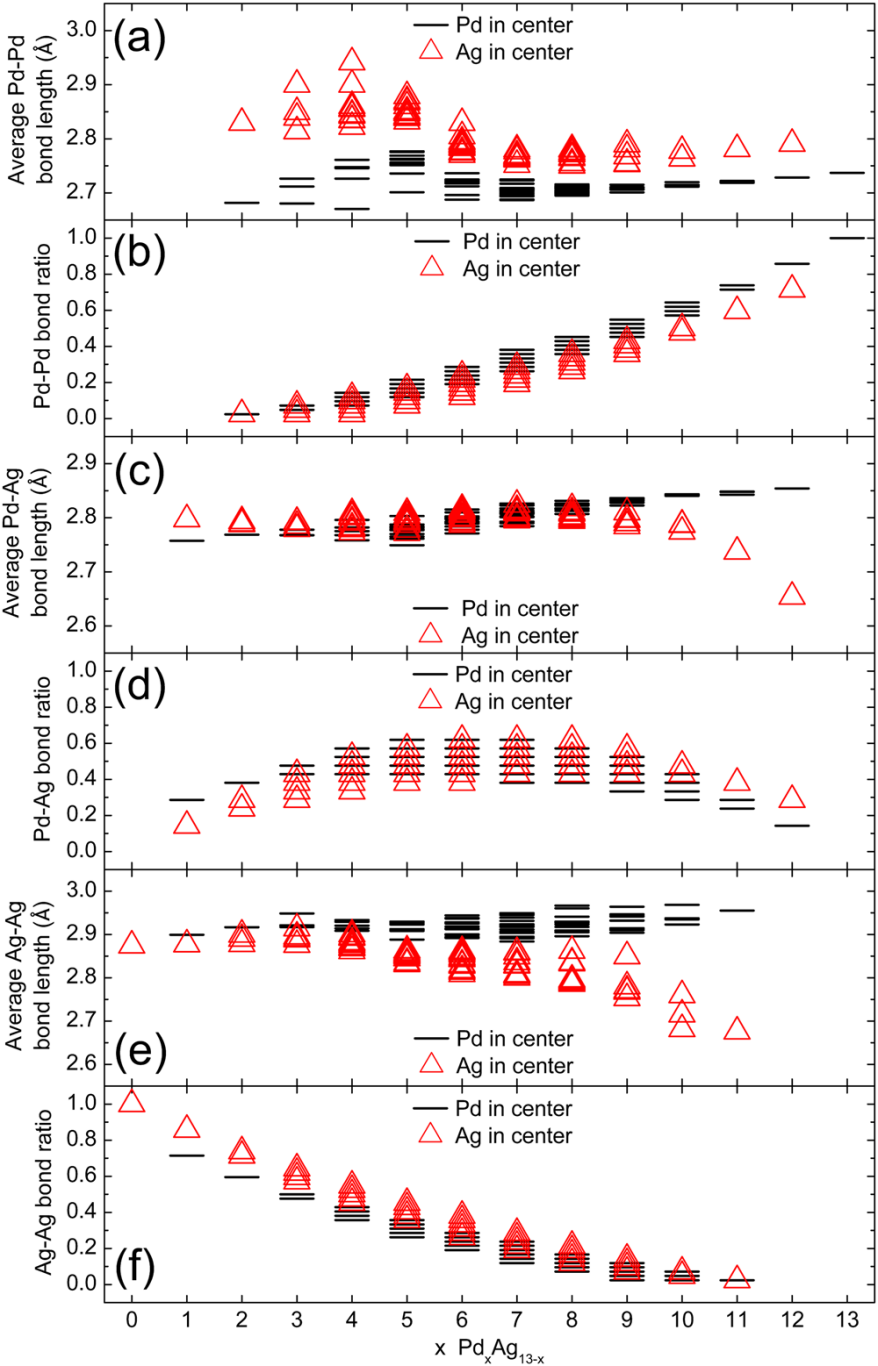
Average bond length (Å) and bond ratio of the Pd_x_Ag_13−x_ (x = 0–13) clusters. (**a**) Average Pd-Pd bond length. (**b**) Pd-Pd bond ratio. (**c**) Average Pd-Ag bond length. (**d**) Pd-Ag bond ratio. (**e**) Average Ag-Ag bond length. (**f**) Ag-Ag bond ratio.

In the icosahedral cluster, there are two kinds of geometric positions, namely the center position (whose coordination number is 12) and the surface position (whose coordination number is 6). The average bond length and bond ratio of Pd-Pd, Pd-Ag and Ag-Ag as a function of x for all the Pd_x_Ag_13−x_, are plotted in Fig. 4(a)–(f), respectively. For the Pd-Pd bond length and bond ratio (Fig. 4(a) and (b)), when the Pd atom is at the center of the cluster, its average bond length is obviously short while its bond ratio is generally large. Also, the Pd-Pd bond ratio gradually increases with increasing x value. Differently, compared with the clusters with central Pd atom, the average Ag-Ag bond length of the clusters with central Ag atom is slightly smaller and decreases sharply when x ≥ 9, as shown in Fig. 4(e). Moreover, the average Ag-Ag bond length of the clusters with central Pd atom displays no obvious difference when the x value changes. Also, the Ag-Ag bond ratio gradually decreases with increasing x value. However, as shown in Fig. 4(c) and (d), the situation is different for the Pd-Ag bimetallic cluster. For the cluster in which the Pd atom is at the center, when x ≤ 6 the average Pd-Ag bond length is generally short, while the Pd-Ag bond ratio is generally large, but when x ≥ 7 the average Pd-Ag bond length is generally large, while the Pd-Ag bond ratio is generally small. For the clusters with a central Ag atom, the average Pd-Ag bond length decreases sharply when x ≥ 9. Additionally, the Pd-Ag bond ratio gradually increases when x ≤ 6, and then decreases slowly when x ≥ 7.

According to the DFT calculations, the Pd-Pd interaction is much stronger than that of Ag-Ag (the binding energy of Pd_13_ is 2.324 eV/atom, while only 1.569 eV/atom for Ag_13_, as listed in Table 1). Moreover, according to the Gupta potential, A_ij_ - B_ij_ in Eq. (3) can reflect the interaction strength. A_ij_ - B_ij_ of Pd-Pd and Ag-Ag are −1.5304 eV and −1.0868 eV, respectively. However, A_ij_ - B_ij_ of Pd-Ag is −1.3990 eV, which is closer to that of Pd-Pd. Accordingly, we suggest that in the Pd-Ag cluster Pd-Pd is strongest, while Pd-Ag is much stronger than Ag-Ag. Whether the Ag or Pd atoms are at the center of the cluster, the binding energy (Fig. 1(a)) quickly increases with increasing x value when x ≤ 5, whereas it increases relatively slowly when x ≥ 5. This is because the Pd-Pd bond ratio always increases with increasing x value but the Pd-Ag bond ratio (Fig. 4(d)) clearly increases with increasing x value when x ≤ 5, whereas it gradually decreases when x ≥ 8.

**Table 1:** Comparison of our calculated values of average bond length (Å), magnetic moment (μB) and binding energy (eV/atom).

	Average bond length		Magnetic moment		Binding energy	
	Ours	ref	ours	ref	ours	ref
Pd_2_	2.470	2.482^c^	2.0	2.0^c^	0.660	0.651^c^
Pd_13_	2.737	2.738^c^	8.0	8.0^c^	2.324	2.313^c^
Ag_2_	2.565	2.59^a^	0	—	0.886	0.87^a^
Ag_13_	2.874	—	5.0	5.0^b^	1.569	1.462^b^

^a^From ref. 3.

^b^From ref. 4.

^c^From ref. 8.

For the Pd-Pd, Pd-Ag and Ag-Ag bonds (Fig. 4(b),(d) and (f), respectively), the bond ratio has no noticeable difference, whether the Ag atom or Pd atom is at the center of the cluster. When x = 1, the average Pd-Ag bond length of the cluster with a central Pd atom is clearly short and the Pd-Ag bond ratio is much higher compared with the clusters with a central Ag atom. In this case, the ΔE_b_ is mainly caused by the Pd-Ag effect, as the Pd-Pd does not exist. In addition, we also notice that the Pd-Ag interaction is much stronger compared with the Ag-Ag interaction because the Pd-Ag bond ratio is clearly smaller than the Ag-Ag bond ratio.

The situation is different when x ≥ 2. On the one hand, the Pd-Ag effect sharply decreases because the average Pd-Ag bond length of the clusters with a central Pd atom displays no evident difference from that of the clusters with a central Ag atom. On the other hand, the Pd-Pd bond starts to appear, and the combined effect of the Pd-Pd and Pd-Ag will impact the stability of the cluster.

Regarding the Ag-Pd cluster in which a different atom is at the center, with increasing x value the difference of the average Pd-Pd bond length decreases when x ≤ 6 and increases when x > 6. Accordingly, ΔE_b_ decreases first and then increases, but the minimum value of ΔE_b_ is at x = 5 due to the gradual increase of the Pd-Pd bond ratio with increasing x value. Whether for Pd-Ag or for Ag-Ag, the average bond length of the clusters with a central Ag atom gradually decreases when x ≥ 8, so that ΔE_b_ increases more slowly, and even decreases at x = 12. This illustrates again that the Pd-Ag interaction is much stronger. Accordingly, we suggest that a strong Pd-Ag interaction should be the reason that the cluster is more stable when the mixability of Pd and Ag atoms is high. In a nutshell, from Fig. 4(a)–(f) we suggest that the large ΔE_b_ (Fig. 1(a)) and its variation are due to much stronger interaction between Pd atoms (Pd-Pd) and between Pd and Ag atoms (Pd-Ag) when the Pd atom is at the center of the cluster.

**Figure 5:**
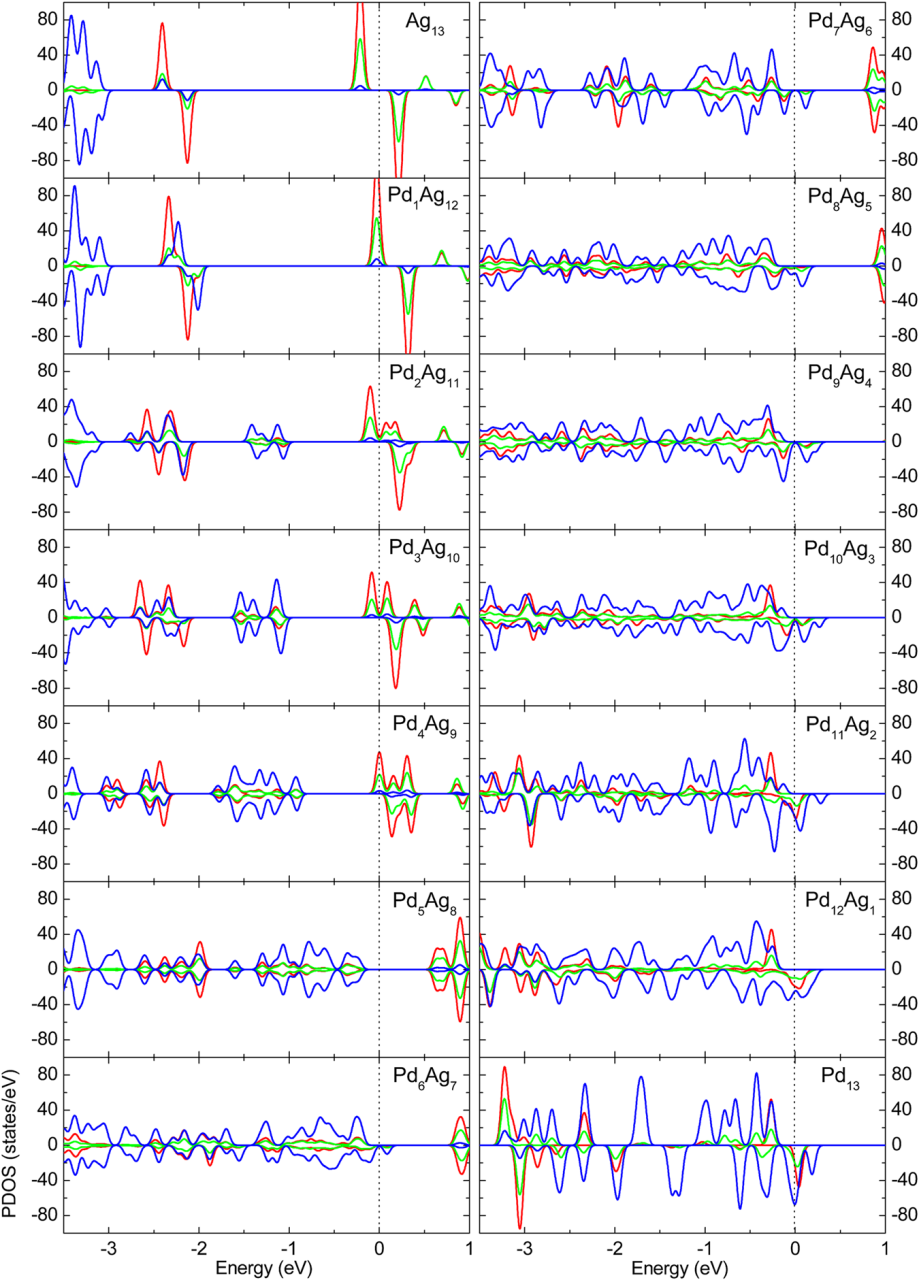
PDOS for each of the most stable structures of the Pd_x_Ag_13−x_(x = 0–13) clusters. Red curve, green curve and blue curve represent *s* × 10, *p* × 10 and *d* states, respectively. Black dashed line indicates the Fermi level.

To further investigate the interactions between Ag and Pd atoms in Pd_x_Ag_13−x_, we plotted the PDOS of the most stable Pd_x_Ag_13−x_ for each x value, as shown in Fig. 5. It is known that the ground state of the Ag atom is 4*d* ^10^5 *s* ^1^. Thus, for Ag_13_, the *s* states are predominant near the Fermi level while the *d* states are far away from the Fermi level (*E*_*F*_). In addition, the states near Fermi level evidently show that Ag_13_ has semiconductor property. However, for Pd_13_, the *d* states are predominant near the Fermi level because the ground state of Pd atom is 4*d* ^10^, and the cluster shows semi-metallic behavior. With the increase of the x value from zero, the amplitude of the states initially decreases and then increases, whilst the *d* states gradually move to the Fermi level.

**Figure 6:**
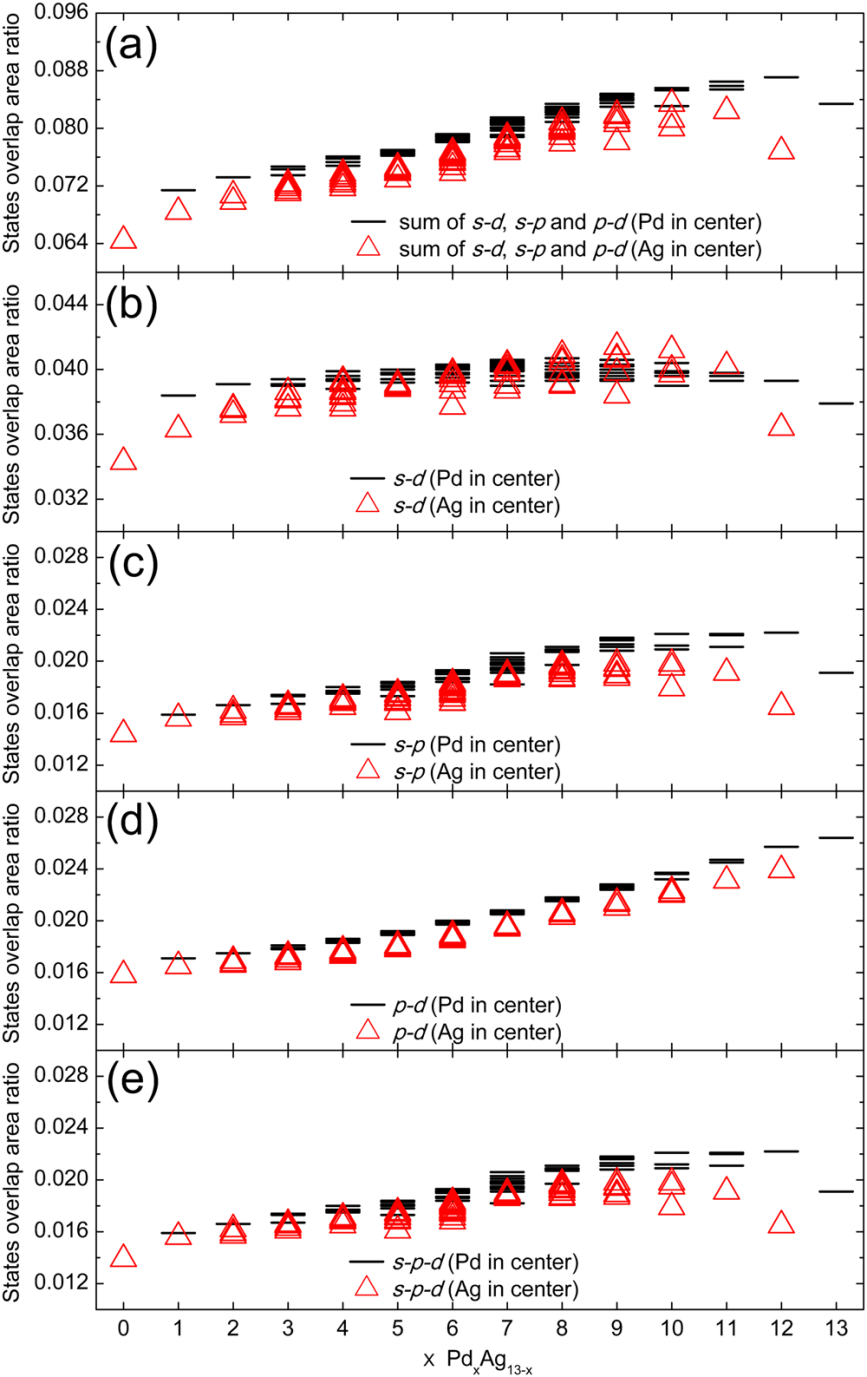
States overlap area ratio of the Pd_x_Ag_13−x_(x = 0–13) clusters. (**a**) Sum of *s*-*d*, *s*-*p* and *p*-*d* states overlap area ratios. (**b**) *s*-*d* states overlap area ratio. (**c**) *s*-*p* states overlap area ratio. (**d**) *p*-*d* states overlap area ratio. (**e**) *s*-*p*-*d* states overlap area ratio.

Previous theoretical studies about the hybridization of Pd clusters have shown that the *s*-*d* hybridization is sensitive to the bond length^6^. In order to illustrate the hybridization in Pd_x_Ag_13−x_ clusters, we calculate the ratio of the *s*-*d*, *s*-*p*, *p*-*d* and *s*-*p*-*d* states overlap area to total states areas of the PDOS below the Fermi energy, as shown in Fig. 6(b)–(e). The sum of the *s*-*d*, *s*-*p* and *p*-*d* states overlap area ratios is shown in Fig. 6(a). It is obvious that the hybridization strength becomes larger with increasing x value. The data in Fig. 6(a)–(e) also reveal that when the Pd atom is at the center of the cluster, the hybridization is stronger. Therefore, hybridization enhancement has a positive effect on the stability of the cluster compared with the binding energies of the clusters (Fig. 1(a)).

**Figure 7:**
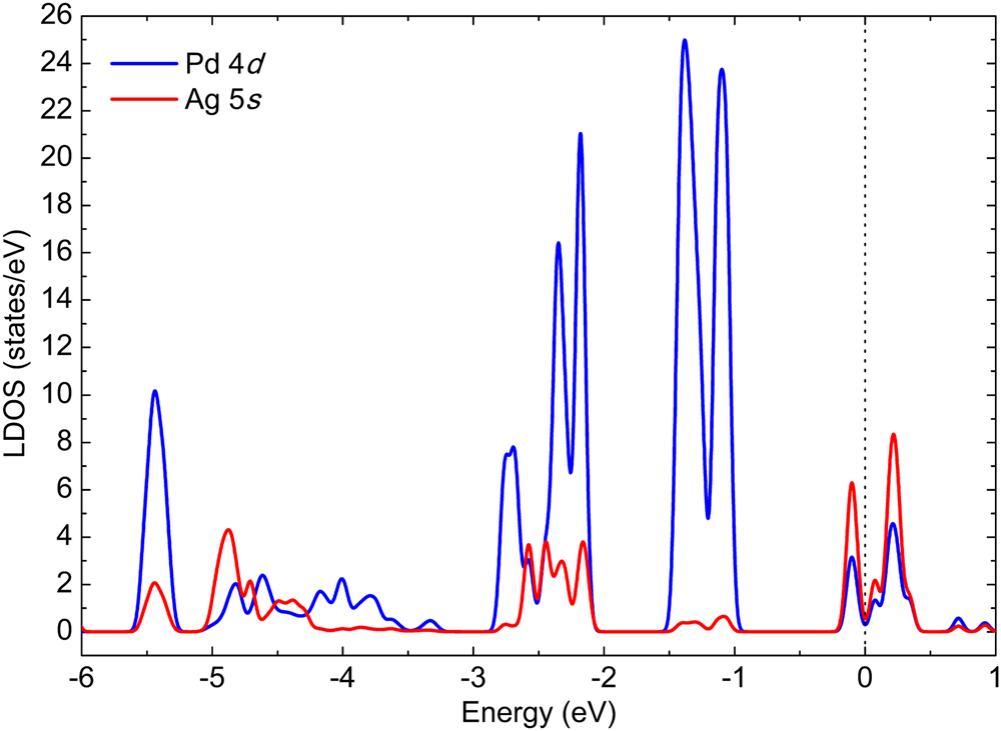
The local density of states (LDOS) originates from the Pd atoms (4*d* orbital) and Ag atoms (5 *s* orbital) of the most stable Pd_1_Ag_12_. Black dot line indicates the Fermi level.

According to the results shown in Fig. 6(a)–(e), for the Pd_x_Ag_13−x_ (x = 0–13) clusters, the *s*-*d* hybridization is predominant. Whether for the clusters with a central Pd atom or for the clusters with a central Ag atom, the *s*-*d* and *s*-*p* hybridization strength increase with increasing x value at the initial stage, but decreases to a certain extent when the x value gets close to x = 13. It is well known that the ground state of the Pd and Ag atoms are 4*d* ^10^ and 4*d* ^10^5 *s* ^1^, respectively. In order to further understand the origin of the strong *s*-*d* hybridization in the Pd-Ag cluster, we calculate its local density of states (LDOS), which shows that when the x value is small the hybridization between Ag atoms (5 *s* orbital) and Pd atoms (4*d* orbital) is much stronger, for example, the LDOS of the most stable Pd_2_Ag_11_ shown in Fig. 7. However, when the x value is large, the electrons of the *s* state in the *s*-*d* hybridization are mainly from the 4*d* electron transitions of Pd atoms, because the number of Ag atoms is small and the *d* state of the Pd atoms is near the Fermi level compared with the PDOS of the Ag_13_ and Pd_13_ shown in Fig. 5. Thus, all the Pd-Ag clusters retain well their icosahedron structure after DFT optimization because of the strong Pd-Ag interaction resulting mainly from much stronger *s*-*d* hybridization.

### Magnetic properties

**Figure 8:**
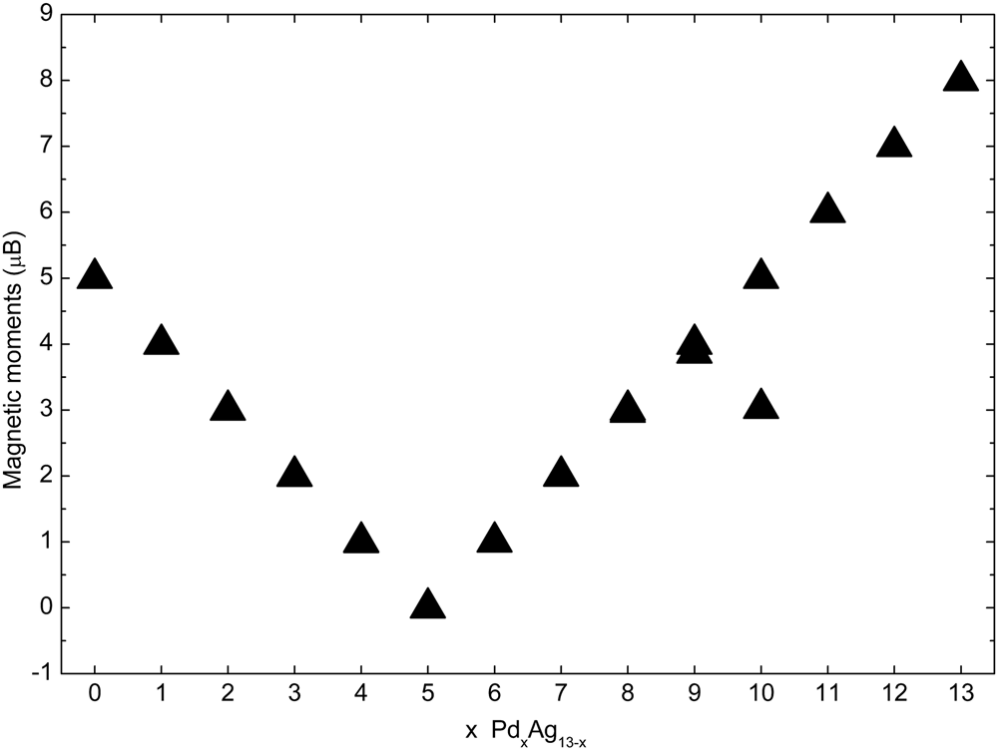
Magnetic moments (μB) of the Pd_x_Ag_13−x_(x = 0–13) clusters.

The magnetic moment for all the icosahedral Pd_x_Ag_13−x_ (x = 0–13) clusters are plotted in Fig. 8. For each x value, all the Pd_x_Ag_13−x_ clusters almost have the same magnetic moment, except for two clusters, i.e., when x = 9 and x = 10. With increasing x value, the magnetic moment linearly decreases from 5.0 μB, until reaching zero at x = 5, and then linearly increases except for those two clusters.

**Figure 9:**
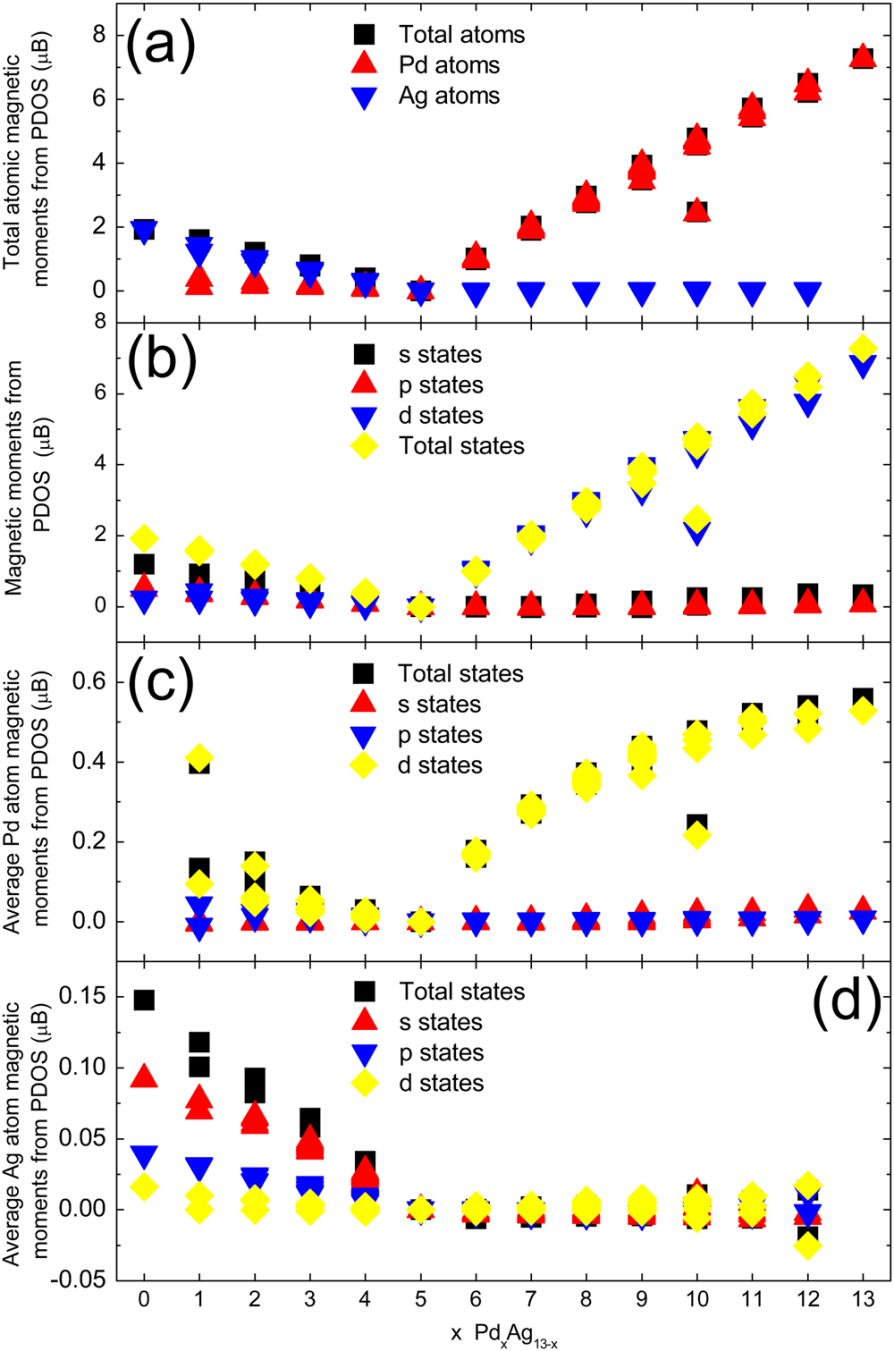
Magnetic moments (μB) of the Pd_x_Ag_13−x_(x = 0–13) clusters calculated from the PDOS. (**a**) Magnetic moments (μB) of the total Pd atoms, total Ag atoms and total atoms. (**b**) Magnetic moments (μB) of the *s*, *p*, *d* and total states. (**c**) Average atomic magnetic moments (μB) of the *s*, *p*, *d* and total states in the Pd atoms. (**d**) Average atomic magnetic moments (μB) of the *s*, *p*, *d* and total states in the Ag atoms.

To understand the variation of the magnetic moments of the present Pd_x_Ag_13−x_ clusters, we calculate the area of PDOS below the Fermi energy (*E*_*F*_), and consider it as the number of electrons (${\int }_{-\infty }^{{E}_{F}}N(E)dE$). The net magnetic moment is equal to the area difference of the PDOS between the spin-up and spin-down below the Fermi energy:

2 $${\int }_{-\infty }^{{E}_{F}}[{N}_{\uparrow }(E)-{N}_{\downarrow }(E)]dE,$$

where *N*(*E*), *N*_↑_(*E*), *N*_*↓*_(*E*) are the total, spin-up and spin-down densities of the states, respectively. Figure 9(a) presents the total Pd and Ag atomic magnetic moment as a function of the x value. It is clear that the magnetic moment mainly originates from Ag atoms when x < 5, while it originates from Pd atoms when x > 5. We also calculate the magnetic moments of *s*, *p*, *d* and total states for all the clusters, as shown in Fig. 9(b). When x < 5, the magnetic moment of the cluster mainly originates from *s* states, while the *d* states are the main contributors to the magnetic moment when x > 5. The average atom magnetic moment (Fig. 9(c) and (d)) evidently shows that the magnetic moment of Ag atoms mostly derives from the unpaired 5 *s* electrons, while the magnetic moment of Pd atoms mostly derives from the incomplete 4*d*-shell electrons.

In detail, hybridization causes the decrease in the number of unpaired 5 *s* electrons in Ag atoms, so that the unpaired 5 *s* electrons contribute to the magnetic moment. In addition, according to the data shown in Fig. 9(c), the *s* states and *p* states of Pd atoms almost do not contribute to the magnetic moment because the electrons of these states come from the 4*d* electrons transitions and mainly participate in hybridization. On the other hand, the ground state of Pd and Ag atoms all have complete 4*d*-shell electrons, and the transition of 4*d* electrons for enhancing hybridization results in incomplete 4*d*-shell. The electrons of the incomplete 4*d*-shell contribute to the magnetic moment according to Hund’s rules. Therefore, whether for Pd atoms or for Ag atoms, larger magnetic moment from the *d* states means more transitions of 4*d* electrons, referring to Fig. 9(c) and (d), respectively. Typically, for Ag_13_, the *d* state makes small contributions to the magnetic moment, indicating that much fewer 4*d* electrons transitions in Ag atoms. In contrast, for the Pd_1_Ag_12_ clusters, the 4*d* electrons transitions of Pd atoms are more evident compared with those of Ag atoms because the transition of the 4*d* electrons in Pd atoms is easier compared with that in Ag atoms.

According to the data in Fig. 9(c) and (d), whether for the *s* states of Ag atoms or for the *d* states of Pd atoms, their contributions to the magnetic moment gradually decreases with increasing x value up to x = 5. We suggest that in the Pd-Ag cluster, the unpaired 5 *s* electrons actually induce the transition of 4*d* electrons, especially for 4*d* electrons of Pd atoms. When x ≤ 5, the unpaired 5*s* electrons quickly decrease with increasing x value, while transitions of 4*d* electrons also decrease, and the magnetic moment is finally quenched at x = 5. When x ≥ 5, as shown in Fig. 9(b)–(d), the electrons of the *s* states almost make no contributions to the magnetic moment, and the magnetic moment derives mainly from the electrons of the *d* states of Pd atoms. Moreover, as shown in Fig. 7, there exists a strong 5*s*-4*d* hybridization between 4 *s* electrons of Ag atoms and 4*d* electrons of Pd atoms. Accordingly, we suggest that the 5*s*-4*d* hybridization between Ag and Pd atoms may also impact the magnetic moment of the Ag-Pd cluster.

## Conclusion

By combining molecular dynamics simulations with a modified Velocity-Verlet algorithm, we have developed an effective global sampling method to extract isomers of bimetallic clusters. Using this method, we obtained isomers of icosahedral Pd_x_Ag_13−x_ (x = 0–13) clusters, all of which retain well the icosahedron structure after DFT optimization because of strong Pd-Ag interaction, mainly from the much stronger *s*-*d* hybridization. When the Pd atom is at the center of the cluster, the Pd_x_Ag_13−x_ is more stable, exhibiting higher binding energy and shorter average bond length. Whether for the clusters with a central Pd atom or for the clusters with a central Ag atom, the clusters are more stable if the degree of mixing of the Pd and Ag atoms is large, which is due to strong Pd-Ag interaction. There exists a large binding energy difference for Pd-Ag cluster because of the strong interaction between Pd atoms (Pd-Pd) and between Pd and Ag atoms (Pd-Ag) when the Pd atom is at the center of the cluster. PDOS calculations showed that the Pd_x_Ag_13−x_ clusters roughly transformed from a semiconductor state to a semi-metallic state when the x value increased from 0 to 13. The icosahedral Pd_x_Ag_13−x_ exhibited magnetic moment except when x = 5. When x < 5, the magnetic moment of the cluster mainly originates from *s* states, while the *d* states are the main contributors to the magnetic moment when x > 5. When x ≤ 5, the unpaired 5 *s* electrons quickly decrease with the increasing number of Pd atoms, while transitions of 4*d* electrons also decrease, so that the magnetic moment is quenched when x = 5. The hybridization has a negative effect on the magnetic moment of Ag atoms, but has positive effect on magnetic moment of Pd atoms.

## Methods

### Sampling method of bimetallic clusters with a given structure

For a given structural n-atom bimetallic A_x_B_n−x_ cluster, there are n!/(x!(n−x)!) geometrical arrangements for each x. To rapidly extract isomers from all geometrical arrangements, we propose a modified Velocity-Verlet algorithm, which can make the cluster system converge rapidly and accurately. We combined this algorithm with molecular dynamics simulations to perform further optimization. By comparing the energy difference, we extracted all the isomers with given composition from the A_x_B_n−x_ clusters. Each of the clusters corresponds to a point in energy axis. We extracted the most stable ones among the optimized clusters falling into each tiny energy interval, and considered them as isomers. All the isomers can be extracted, provided the energy interval is small enough. During the extraction of the icosahedral Pd_x_Ag_13−x_ (x = 0–13) clusters, we applied the Gupta potential using the tight-binding scheme to calculate the energy of cluster^10^.

### Gupta potential

The Gupta potential is a many-body characteristic potential, which contains a repulsive term E^r^(i) and an attractive term E^a^(i). Energy of atom i can be expressed as:

3 $${{\rm{E}}}^{{\rm{r}}}({\rm{i}})+{{\rm{E}}}^{{\rm{a}}}({\rm{i}})={\rm{\sum }}_{{\rm{i}}{\rm{\ne }}{\rm{j}}}^{{\rm{N}}}{{\rm{A}}}_{{\rm{i}}{\rm{j}}}{{\rm{e}}}^{-{{\rm{P}}}_{{\rm{i}}{\rm{j}}}({{{\rm{r}}}_{{\rm{i}}{\rm{j}}}/{\rm{R}}}_{{\rm{i}}{\rm{j}}}-1)}{\rm{-}}\sqrt{{\rm{\sum }}_{{\rm{i}}{\rm{\ne }}{\rm{j}}}^{{\rm{N}}}{{\rm{B}}}_{{\rm{i}}{\rm{j}}}^{{\rm{2}}}{{\rm{e}}}^{-{\rm{2}}{{\rm{Q}}}_{{\rm{i}}{\rm{j}}}({{\rm{r}}}_{{\rm{i}}{\rm{j}}}/{{\rm{R}}}_{ij}-{\rm{1}})}}$$

where r_ij_ is the distance between atoms i and j, R_ij_ is the equilibrium distance, parameters A_ij_, B_ij_, P_ij_, Q_ij_ and R_ij_ depend on the type of bond, and these parameters for Pd-Ag, Pd-Pd and Ag-Ag are listed in Table 2.

**Table 2:** Parameters of the Gupta potential^10^.

	A_ij_ (eV)	B_ij_ (eV)	P_ij_	Q_ij_	R_ij_ (Å)
Pd-Pd	0.1715	1.7019	11.0	3.794	2.75
Ag-Ag	0.1031	1.1899	10.85	3.18	2.89
Pd-Ag	0.1607	1.5597	10.895	3.492	2.82

### Modified Velocity-Verlet algorithm

Both the force and energy will greatly change when the atomic distance is close to the equilibrium distance. During the molecular dynamics simulations for the clusters, a standard Velocity-Verlet algorithm is usually used^13^. If the time step is set too small, the simulations will consume massive computational time. However, if the time step is set too large, it will lead to irrational structure disruption. Therefore, we made two amendments to the standard Velocity-Verlet algorithm. Position and velocity of particle i can be calculated from the following formulas:

4 $${{\bf{r}}}_{{\rm{i}}}({\rm{t}}+{\rm{\Delta }}{\rm{t}})={{\bf{r}}}_{{\rm{i}}}({\rm{t}})+{\rm{\Delta }}t\cdot {{\bf{V}}}_{{\rm{i}}}({\rm{t}})+0.5\cdot {({\rm{\Delta }}{\rm{t}})}^{2}\cdot {{\bf{F}}}_{{\rm{i}}}({\rm{t}})/{\rm{m}}$$

5 $${{\bf{V}}}_{{\rm{i}}}({\rm{t}}+{\rm{\Delta }}{\rm{t}})={{\bf{V}}}_{{\rm{i}}}({\rm{t}})+0.5\cdot {\rm{\Delta }}t\cdot [{{\bf{F}}}_{{\rm{i}}}({\rm{t}})+{{\bf{F}}}_{{\rm{i}}}({\rm{t}}+{\rm{\Delta }}t)]/{\rm{m}}$$

where m is the mass of the particle; **F**_i_(t) is the atomic force, which is an energy gradient **F**_i_(t) = −∇E_i_(t). We replaced **V**_i_(t + Δt) by **V**_i_(t + Δt)·e^−j·ETA^ at the end of each iteration, i.e.

6 $${{\bf{V}}}_{{\rm{i}}}({\rm{t}}+{\rm{\Delta }}t)\to {{\bf{V}}}_{{\rm{i}}}({\rm{t}}+{\rm{\Delta }}t)\cdot {{\rm{e}}}^{-{\rm{j}}\cdot {\rm{ETA}}}$$

where j is the present iteration, the modifying factor e^−j·ETA^ can be regarded as the reflex of the damping effect, and ETA is a constant representing the intensity of the damping effect. A simple model of the damping effect can be expressed as:

7 $${\bf{F}}=-{\rm{k}}\cdot {\bf{V}}$$

where k is a constant. From Newton’s laws of motion, we obtain

8 $${\bf{V}}({\rm{t}}+{\rm{\Delta }}t)={\bf{V}}({\rm{t}})\cdot {{\rm{e}}}^{-k\cdot }{{}^{{\rm{\Delta }}}}^{{\rm{t}}/{\rm{m}}}$$

In Eq. (6), e^−k·Δt/m^ is a constant. However, the modifying factor e^−j·ETA^ in Eq. (6) gradually enlarges the attenuation of velocity with the passage of time. It makes the system evolve in the former period by the standard Velocity-Verlet algorithm and converge in the end. On the other hand, we set a cut-off force (F_max_) to prevent irrational structure disruption caused by too large time step. However, we did not change the direction of force.

**Figure 10:**
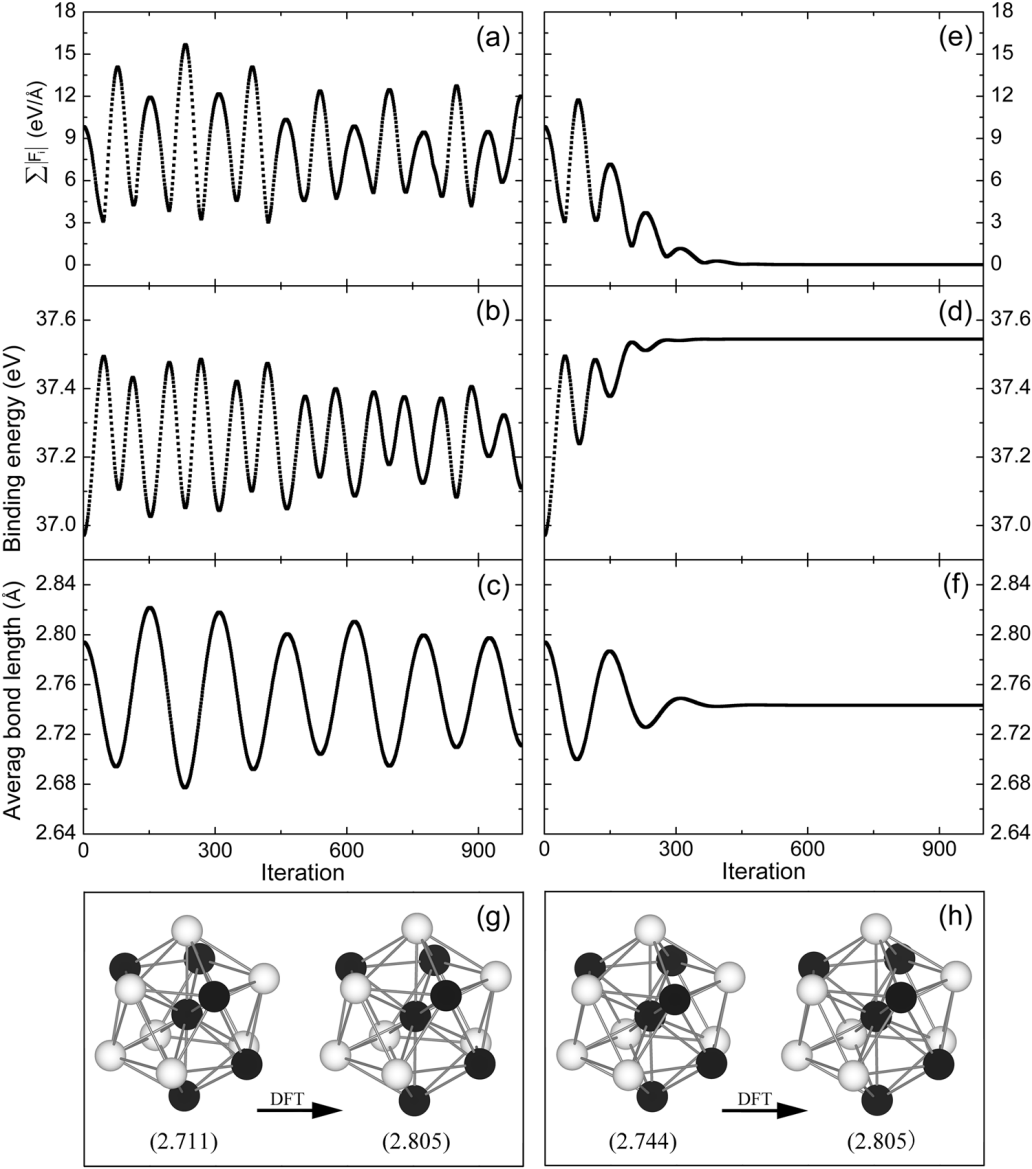
Sum of the atomic absolute force ∑|F_i_| (eV/Å), binding energy (eV) and average bond length (Å) of the Pd_6_Ag_7_ calculated with the standard Velocity-Verlet algorithm (**a**–**c**), and the modified Velocity-Verlet algorithm (**e**)–(**f**). Sum of atomic absolute forces in (**a**) is before being cut off. (**g**) The structure of Pd_6_Ag_7_ obtained using the standard Velocity-Verlet algorithm (left) and then optimized by the DFT calculations (right). (**h**) The structure of Pd_6_Ag_7_ obtained with the modified Velocity-Verlet algorithm (left) and then optimized by the DFT calculations (right). The black and white spheres represent the Pd and Ag atoms, respectively. The values in the brackets of (**g**) and (**h**) are the average bond lengths (Å) of Pd_6_Ag_7_.

In order to test the reliability and accuracy of our modified Velocity-Verlet algorithm for the optimization of clusters, we chose the Pd_6_Ag_7_ cluster to carry out the calculations. The initial Pd_6_Ag_7_ cluster was constructed by directly substituting six Ag atoms with Pd atoms in an optimized icosahedral Ag_13_ cluster. We set ETA = 0.0001, Δt = 0.01, m = 1.0, and F_max_ = 10 eV/Å. The iteration was set to 1000 and the initial velocities of all atoms were set to zero. We introduced them into Eqs (4–6). The ∑|F_i_|, which is the sum of atomic absolute force before being cut off, is shown in Fig. 10(e). The binding energy and the average bond length of the cluster at different iterations, are plotted in Fig. 10(d) and (f), respectively. These values converge well when the iteration is more than 500, indicating that our modification can make the system converge rapidly and accurately. We also calculated these characteristic values using the standard Velocity-Verlet algorithm, as shown in Fi﻿g. 10(a)–(c). In comparison with Fig. 10(e)–(f), it is clear that our modified Velocity-Verlet algorithm can make the cluster converge more rapidly and accurately. The structures of Pd_6_Ag_7_ obtained by these two algorithms and subsequently optimized by DFT calculations are presented in Fig. 10(g) and (h). After optimization by DFT calculations, the final cluster structures generated by these two algorithms are almost the same, and both have the same average bond length (2.805 Å). In addition, the average bond length of Pd_6_Ag_7_ obtained by our modified algorithm is closer to the result from the DFT optimization. Furthermore, it can quickly tend to a stable value even if the DFT calculation time is greatly reduced. Thus, the above tests indicate that our modified algorithm is not only reliable, but also more efficient and more accurate than the standard Velocity-Verlet algorithm.

During the extraction of the icosahedral Pd_x_Ag_13−x_ (x = 0–13) clusters, we directly substituted x Ag atoms with Pd atoms in the optimized icosahedral Ag_13_ cluster to obtain the initial structures of the clusters which have 13!/(x!(13﻿–x)!) geometrical arrangements. All the molecular dynamics simulations are based on our modified Velocity-Verlet algorithm, whose parameters are ETA = 0.0001, Δt = 0.01, m = 1.0 and F_max_ = 10 eV/Å. The initial velocities of all atoms were set to zero. The tiny energy interval and iteration were set as 0.0001 eV and 2000, respectively, which are sufficient to ensure the energy convergence accuracy and distinguish isomers of the Pd-Ag clusters.

### Computational details of DFT calculations

Based on the optimized structures obtained by the modified molecular dynamics simulations, we performed further structural optimization using the first-principle spin-polarized DFT method. The Vienna ab initio simulation package (VASP) was used^14^ and the projector augmented wave (PAW) was implemented^15, 16^. The exchange-correlation functional was described by the Perdew, Burke, and Ernzerhof (PBE) functional within the generalized gradient approximation (GGA)^17^. The plane-wave basis was set with an energy cut-off of 550 eV. The equilibrium geometries are obtained when the atomic forces are smaller than 0.01 eV/Å and the total energy convergences are within 10^−5^ eV. All calculations were performed within a cubic box of 20 Å and using a single k point (Г point) for the Brillouin-zone (BZ) integration.

The accuracy of the DFT calculations was assessed by calculating Pd_2_ dimer, Ag_2_ dimer, Pd_13_ and Ag_13_ icosahedral clusters. For each of these clusters, our calculated average bond length, binding energy and magnetic moment agree well with previous computational values listed in Table 1, indicating that our calculated method in this work is reliable.
